# National genomic evaluation for a novel functional longevity trait

**DOI:** 10.1186/s12711-026-01087-0

**Published:** 2026-09-18

**Authors:** Pedro Ramos, Shogo Tsuruta, Andre Garcia, Kelli Retallick, Stephen P. Miller, Daniela Lourenco

**Affiliations:** 1 Angus Genetics Inc, Saint Joseph, MO USA; 2 Department of Animal and Dairy Science, University of Georgia, Athens, GA USA; 3 AGBU, A Joint Venture of NSW Department of Primary Industries and Regional Development, University of New England, Armidale, Australia

## Abstract

**Background:**

Cow longevity plays a major role in beef cattle industry profitability, reflecting the female’s ability to remain productive in the herd. In this context, longitudinal models as random regression models (RRM) are the preferred method for genetic evaluation as they account for genetic and environmental effects over time. Traditionally, RRM longevity evaluations use binary phenotypes; however, redefining the phenotype as the number of calves could enhance variability, better identify cows with consistent reproductive performance, and penalize irregular calving patterns. To address these limitations, this study aimed to: (1) propose genomic evaluations for a novel functional longevity (FL) definition via RRM; (2) compare model fit using Legendre polynomials (LP) of varying orders; (3) evaluate the implication of FL genetic parameters for selection strategies.

**Results:**

The dataset included records from 2 million Angus cows and 1.6 million genotyped animals from the American and Canadian Angus Associations. FL was defined as the cumulative number of calvings, measured annually from 2 to 10 years of age. Genetic parameters were estimated with Gibbs sampling without genomic information, and genomic breeding values (GEBV) were estimated using RRM under single-step GBLUP with LP. Models were compared via the deviance information criterion and sire GEBV rankings across ages were evaluated using Spearman correlations and percentage of individuals in common (top 1%). The best fit was with a third-order LP and homogeneous residual variance. Heritabilities ranged from 0.04 to 0.12. Genetic correlations between ages were positive and high, with minimal fluctuations. GEBV rank correlations were consistent across ages, with Spearman correlations following the genetic correlations pattern. The percentage of commonly selected individuals between adjacent years was high. However, percentage decreased with increasing age gap, reaching 53% between ages 3 and 10.

**Conclusions:**

The new functional longevity definition is feasible for genomic evaluations and has a straightforward interpretation as breeding values represent the number of additional calves when comparing animals. FL heritability estimates indicate sufficient additive genetic variance to allow moderate response to selection. Based on genetic correlations and ranking comparisons, functional longevity at 6 years of age is the optimal age to maximize genetic progress in Angus cattle.

## Background

Reproductive efficiency significantly influences the productivity and profitability of beef cattle production systems [1]. Additionally, reproductive traits greatly affect the generation interval and selection intensity [2], thereby influencing the rate of genetic progress over time. Consequently, various female reproductive traits have been evaluated as selection criteria in beef cattle breeding programs worldwide. Among these traits, longevity plays a critical role, as it reflects a female’s ability to remain in the herd up to a certain age while maintaining consistent reproductive performance [3].

Several definitions of longevity have been proposed over time, considering the time cows remain in the herd and the productivity throughout their lives. The most common measure of longevity is considered a binary trait evaluated by a threshold model, in which success is defined as a cow achieving a significant number of calvings by a certain age, such as 6 years [4, 5]. However, this definition restricts the number of cows included in evaluations, as young females cannot be assessed until they reach the target age. The lack of phenotypic data for young females can reduce the accuracy of estimated breeding values (EBV) for this important trait and for young sires, whose EBV accuracy depends partly on their daughters’ contributions [6]. Furthermore, this traditional definition may fail to differentiate between cows with varying numbers of calvings, as any cow reaching the target number is considered successful, regardless of differences in reproductive performance (i.e., cows can fail consecutive breeding seasons and still remain in the herd). Additionally, this approach does not account for the timing of reproductive failures. Cows with different numbers of calvings and failures occurring at different points in their lives can end up with the same phenotype, limiting the ability to accurately capture variations in their reproductive performance.

Therefore, developing new methodologies to better define cow reproductive performance and more accurately predict breeding values is crucial for selecting more efficient animals. In this context, longevity can be analyzed longitudinally using random regression models (RRM) [3]. Unlike traditional models, which use a single record per cow, RRM utilize records from multiple time points and account for random genetic and environmental effects over time. In addition, RRM can model the covariance pattern between the yearly records [7]. Thus, RRM can be a feasible alternative to increase the accuracy of breeding values for longevity and evaluate genetic parameters as a function of the evaluation period.

The most common approach for evaluating longevity using a random regression model involves also treating the phenotype at each cow’s age as binary records [3], where the phenotype for each age is classified as a success if the cow produces a calf and a failure if she does not. In this case, the EBV are predicted on a liability scale and converted into probabilities [8]. For functional longevity, this represents the probability of a cow calving at each specific age, providing a more interpretable metric for genetic selection. However, the probability of calving at a specific age may not effectively capture cows with better parturition regularity across multiple years. To address this limitation, some studies have used binary records to define the phenotype as the probability of a cow producing consecutive calvings up to each age of the evaluation period [9, 10]. In this second approach, if a cow fails to calve in a given year, she is assigned a failure or a missing phenotype for subsequent ages. This method, however, disregards calvings that occur after a reproductive failure, potentially treating cows with varying numbers of non-consecutive calvings as having the same phenotype. Veerkamp et al. [11] applied random regression models to censored survival data, coding survival as binary outcomes and treating incomplete records as censored observations. In such models, the likelihood must accommodate both observed events and non-events, which substantially increases computational demands, particularly when likelihood-based integration methods are used. Although feasible for smaller datasets, this added complexity becomes a major challenge in large-scale commercial evaluations involving millions of records. In this context, modeling reproductive output and treating post-exit records as biologically meaningful missing data, rather than censored observations, offers a practical alternative. This approach allows discontinuities to be explicitly represented, improving both computational efficiency and the interpretability of genetic trajectories.

Therefore, defining the phenotype as the cumulative number of calves a cow has produced up to each age, rather than as a simple binary success/failure record, could improve the effectiveness of random regression models in identifying the most productive cows and enhancing the interpretation of Expected Progeny Differences (EPD). This method would increase phenotype variability, better identify cows with consistent reproductive performance over time, and penalize those with irregular calving patterns.

In this context, our objectives were to: (1) propose a National North American Angus Genomic Evaluation of a new functional longevity definition based on the cumulative number of calvings using random regression models; (2) compare the goodness-of-fit of RRM employing orthogonal Legendre polynomials of varying orders; and (3) evaluate the behavior of genetic parameters for functional longevity over time and their implications for selection strategies.

## Methods

The datasets were provided by the American Angus Association (Saint Joseph, MO, USA) and the Canadian Angus Association (Rocky View County, AB, Canada). The datasets were merged after preliminary analyses indicated that the data from the USA and Canada could be integrated into a single genomic evaluation (results not shown). The functional longevity trait was analyzed longitudinally, defined as the cumulative number of calvings a cow had throughout her lifetime, measured annually over a 9-year evaluation period (from 2 to 10 years of age). A penalization was applied to cows that remained in the herd but failed to calve. In such years, the cow’s phenotype was assumed to be unchanged from the previous year, ensuring that periods of non-calving were accurately incorporated into the analysis.

**Fig. 1:**
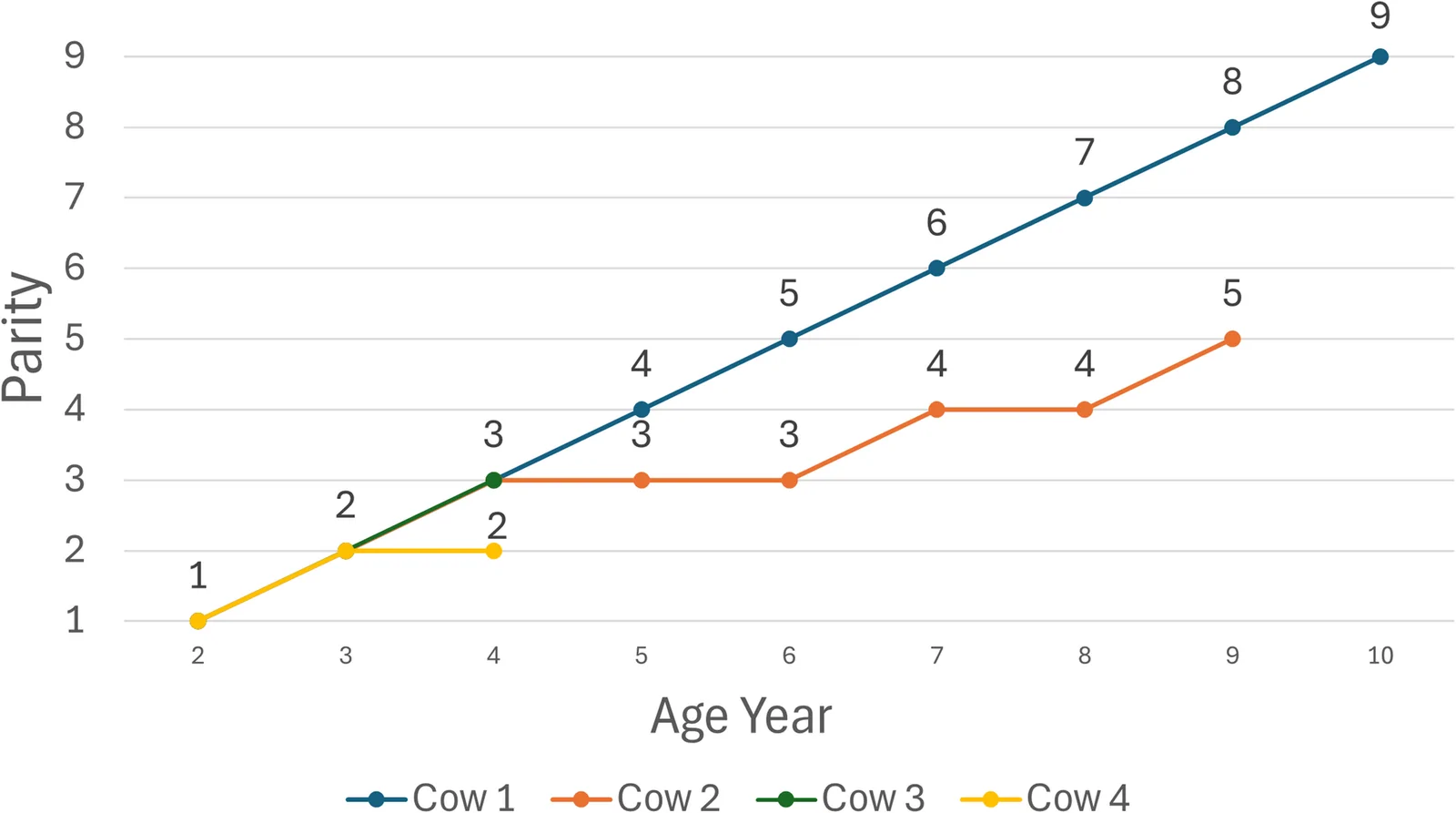
Functional longevity phenotype definition. Legend. Example of phenotype definition across ages for four cows with varying numbers of calvings throughout their lifetime

The cumulative calving count is not a repeated measure of the same event, but rather a longitudinal accumulation of distinct reproductive outcomes. Each year’s phenotype reflects a new biological opportunity, either realized (a calving) or missed (no calving). The covariance structure between ages is explicitly modeled via the additive genetic covariance matrix (Σₐ), permanent environmental effects (Σₚ), and residual variance components. This framework ensures that age-specific records are not treated as independent, genetic correlations between ages are estimated and incorporated, and the model captures the smooth trajectory of reproductive performance, not discrete jumps. Biologically, a missed calving is not a transient event, it has cumulative consequences. It delays the next calving, reduces the total number of calves over a cow’s lifetime, and may signal underlying fertility. Figure 1 illustrates the definition of the functional longevity (FL) phenotype, providing an example of four cows with varying numbers of calvings and penalizations throughout their lifetime.

Only cows with calving records at two years of age were included in the analyses. Calving records for cows aged 11 years and older were excluded due to the limited amount of data available. The reduced number of records in these age categories is a consequence of the selection process and may cause instability in the variance components, particularly at the extremes of the curve, where overfitting can occur (Runge’s phenomenon) [12, 13]. Additionally, cows born before 1990 or those that did not have their first calving by 33 months of age were removed from the dataset. The minimum age considered for the first calving was 18 months. Records were considered missing for ages when cows were no longer in the herd due to death or culling. The timing and reason of culling was determined based on information provided by breeders through recording a disposal code. For cows without culling data, it was assumed they were culled after three years without producing a calf. In such cases, it was inferred that they were removed from the herd after failing to conceive during the first year of the three-year period.

Contemporary groups (CG) were defined based on herd, birth year, and the season (1 = March to May, 2 = June to August, 3 = September to November, and 4 = December to February) of the cow’s first calf. The CG effect is fitted as a fixed regression adjusting for the average trend over the ages. Additionally, a second effect was assumed to account for the group within age. This second effect, referred to as the calf contemporary group, was determined by the birth year and season of the calf at each specific age. Animals belonging to CG with less than five records were excluded. The pedigree file included the animals of the last three generations from animals with phenotypes and/or genotypes. The complete dataset (DATA_FL) included 8,525,448 phenotypic records from 2,011,569 Angus cows. The genotype files contained 1,612,618 animals genotyped for 39,685 SNPs after quality control, and the complete pedigree file contained 4,243,224 animals. A subset (DATA_sub) was created for variance components estimation (VCE) by randomly selecting contemporary groups. Because CG do not change over time, when sampled all records from cows in those CG were included in the subset. In that, there was a total of 1,118,616 records from 245,351 cows, and the number of animals in the pedigree was 666,059. No genomic information was used for VCE. Descriptive statistics of the datasets are presented in Table 1.

**Table 1:** Number of records, mean, and standard deviations (SD) for Functional Longevity in each dataset

			Number of Calvings per Age											
	AGE	*N*	0	1	2	3	4	5	6	7	8	9	MEAN	SD
DATA_FL	2	2,011,569	0	2,011,569	0	0	0	0	0	0	0	0	1.00	0.00
	3	1,826,033	3564	489,268	1,333,201	0	0	0	0	0	0	0	1.73	0.45
	4	1,370,718	2414	55,735	421,336	891,233	0	0	0	0	0	0	2.61	0.58
	5	1,039,597	2561	4,035	106,391	313,337	613,273	0	0	0	0	0	3.47	0.71
	6	777,088	2197	225	18,169	98,831	230,936	426,730	0	0	0	0	4.36	0.83
	7	577,031	1620	64	1,818	25,315	80,895	171,790	295,529	0	0	0	5.26	0.93
	8	422,105	1264	41	192	5,055	24,649	63,134	126,546	201,224	0	0	6.15	1.03
	9	300,747	904	4	43	833	6,520	20,806	47,866	91,094	132,677	0	7.05	1.13
	10	200,560	805	0	9	38	1,088	5,278	14,356	31,746	63,435	83,805	7.98	1.20
	Total	8,525,448	15,329	2,560,941	1,881,159	1,334,642	957,361	687,738	484,297	324,064	196,112	83,805	-	-
DATA_SUB	2	245,310	0	245,310	0	0	0	0	0	0	0	0	1.00	0.00
	3	235,849	96	55,816	179,937	0	0	0	0	0	0	0	1.76	0.43
	4	190,002	122	6249	53,977	129,654	0	0	0	0	0	0	2.65	0.55
	5	144,037	112	347	11,800	43,341	88,437	0	0	0	0	0	3.52	0.66
	6	106,661	75	24	1731	11,905	32,171	60,755	0	0	0	0	4.42	0.76
	7	77,722	79	1	136	2665	10,017	23,831	40,993	0	0	0	5.32	0.86
	8	55,424	59	0	10	491	2629	7894	17,291	27,050	0	0	6.22	0.95
	9	38,442	34	0	0	62	604	2322	5906	12,333	17,181	0	7.11	1.02
	10	25,169	33	0	0	2	92	506	1581	3883	8633	10,439	8.05	1.07
	Total	1,118,616	610	307,747	247,591	188,120	133,950	95,308	65,771	43,266	25,814	10,439	-	-

N, Total number of records; SD, standard deviation; Data_FL, Phenotypic data of Functional Longevity; DATA_SUB, subset data of Functional Longevity used for variance component estimation

### Statistical analyses

The variance components and genetic parameters were estimated using a random regression model applied to the Data_sub, with Bayesian methods based on Gibbs sampler and Markov Chain Monte Carlo (MCMC) algorithm, implemented through the GIBBS3f90 software [14]. The systematic herd-year-season effect and the random additive genetic and permanent environmental effects were modeled using orthogonal Legendre polynomials of orders one to three, with homogeneous and heterogeneous residual variances. Different residual variances for each age were also evaluated. The most suitable model was selected by comparing the goodness of fit using the deviance information criterion (DIC), and a validation analysis using the linear regression method [LR;15]. The LR method was applied using a set of focal individuals composed of all animals born after 2018, totaling 319,894 out of 2,106,305 individuals in the complete dataset. For each model, breeding values were estimated twice: first using the whole dataset (w), which included phenotypic records from the focal individuals, and then using a partial dataset (p), excluding their phenotypes. Model performance was evaluated based on the estimated bias and prediction accuracy for the focal individuals as described in the equations below [15]:

1 $$\:\widehat{\mathrm{a}\mathrm{c}\mathrm{c}}=\sqrt{\frac{\mathrm{c}\mathrm{o}\mathrm{v}({\widehat{\mathrm{u}}}_{\mathrm{w}},{\widehat{\mathrm{u}}}_{\mathrm{p}})}{(1-\overline{\mathrm{F}}){\widehat{{\upsigma\:}}}_{\mathrm{u}}^{2}}}$$

where $\:{\widehat{u}}_{w}$ and $\:{\widehat{u}}_{p}$ are the vectors of breeding values estimated for the candidates in the whole and partial datasets, respectively, $\:\overline{F}$ is the average inbreeding of the candidates, and $\:{\widehat{\sigma\:}}_{u}^{2}$ is the additive genetic variance.

Bias:

2 $$\:{{\upmu\:}}_{\mathrm{w}\mathrm{p}}=\:{\overline{\widehat{\mathrm{u}}}}_{\mathrm{p}}-{\overline{\widehat{\mathrm{u}}}}_{\mathrm{w}}$$

To reduce the number of comparisons and maintain model parsimony, the same polynomial order was used to describe the curves of the three effects in each compared scenario [9]. Additionally, genetic correlations between different years of a cow’s life were estimated using the genetic (co)variances between each age.

The Bayesian random regression model can be represented in matrix notation as:

3 $$\mathbf{y} = \mathbf{X}\mathbf{b} + \mathbf{Z}\mathbf{a} + \mathbf{W}\mathbf{p} + \mathbf{e}$$

where **y** is the vector of observations (Number of calvings), with $$\mathbf{y} \mid \mathbf{b},\mathbf{q},\mathbf{a},\mathbf{p},\mathbf{G}_a,\mathbf{R}_p,\sigma_e^2\sim N$$$$\left(\mathbf{X}\mathbf{b} + \mathbf{K}\mathbf{q} + \mathbf{Z}\mathbf{a} + \mathbf{W}\mathbf{p},\,\mathbf{I}\sigma_e^2\right)$$, in which$$\:{{\upsigma\:}}_{\mathrm{e}}^{2}$$ is the residual variance; **b** is the vector of systematic effects, including a categorical variable that indicates whether the cow was born through embryo transfer and the calving year season, along with the coefficients of fixed regression for the herd-year-season effect (average regression for each herd-year-season of birth), assuming a uniform prior distribution; **a** is the vector of regression coefficients for the additive genetic effect, $$\:\mathbf{a}|{\boldsymbol{\Sigma\:}}_{\boldsymbol{a}},\:$$$$\:\mathbf{A}$$$$\:\:\sim\:N\:(0,\:{\boldsymbol{\Sigma\:}}_{\boldsymbol{a}}\otimes\:$$ $$\:\mathbf{A}$$* )*, in which**A** is the pedigree relationship matrix, and $$\:{\boldsymbol{\Sigma\:}}_{\boldsymbol{a}}$$ is the additive genetic covariance matrix; **p** is the vector of regression coefficients for the random permanent environmental effect, assuming $$\:\mathbf{p}|{\boldsymbol{\Sigma\:}}_{\boldsymbol{p}}\sim\:N\:(0,\:{\boldsymbol{\Sigma\:}}_{\boldsymbol{p}}\otimes\:\mathbf{I})$$, which **I** is an identity matrix and $$\:{\boldsymbol{\Sigma\:}}_{\boldsymbol{p}}$$ is a matrix of permanent environmental variance. The incidence matrices for **b**, **a**, and **p**, are **X**, **Z**, and **W**, respectively.

A total of 450,000 MCMC iterations, considering a burn-in period of 200,000 and a sampling interval (thin) of 10 cycles were used. The POSTGIBBSF90 software [14] was used for posterior inference, and the convergence was evaluated graphically and through the Geweke test [16].

The genomic estimated breeding values (GEBV) for each age were calculated using the complete functional longevity dataset (DATA_FL) with the blup90iod2OMP1 software [14], under the single-step genomic BLUP (ssGBLUP) methodology. In ssGBLUP, the covariance is given by the realized relationship matrix (**H**), which combines pedigree and genomic relationships [17].

According to Aguilar et al. [18], the inverse of **H** ($\:{\mathrm{H}}^{\mathrm{-1}}$) is given by:

4 $${\mathbf{H}}^{-1}={\mathbf{A}}^{-1}+\left[\begin{array}{cc}0&0\\0&{\mathbf{G}}^{-1}{\mathbf{A}}_{22}^{-1}\end{array}\right]$$

where $\:{\mathbf{A}}_{22}^{-1}$ is the inverse of the pedigree relationship matrix for genotyped animals; $\:{\mathbf{G}}^{-1}$ is the inverse of the genomic relationship matrix ($\:\mathbf{G}$) constructed based on the type I described by VanRaden [19]:

5 $$\:\mathbf{G}=\frac{\mathbf{M}\mathbf{M^\prime}}{2\sum\:{\mathrm{p}}_{\mathrm{i}}(1-{\mathrm{p}}_{\mathrm{i}})}$$

where **M** = $\:{\mathbf{M}}_{0}$ − **P**, being $\:{\mathbf{M}}_{0}$ a matrix of SNP content for each animal and **P** is a matrix of twice the allele frequency of the second allele p at locus i ($\:{\mathrm{p}}_{\mathrm{i}}$). To avoid singularity, 90% of **G** was blended with 10% of **A**_22_. Because of the large number of genotyped animals in DATA_FL, the algorithm for proven and young (APY) [20] was applied to obtain a sparse representation of $\:{\mathbf{G}}^{-1}$ without having to invert **G** directly. Therefore, $\:{\mathbf{G}}^{-1}$ in Eq. (4) was replaced by the $\:{\mathbf{G}}_{\mathbf{A}\mathbf{P}\mathbf{Y}}^{-1}$ constructed as below:

6 $$G_{\mathrm{APY}}^{-1}=\begin{bmatrix}G_{cc}^{-1} & 0 \\0 & 0\end{bmatrix}+\begin{bmatrix}-G_{cc}^{-1}G_{cn} \\I\end{bmatrix}M^{-1}\begin{bmatrix}-G_{nc}G_{cc}^{-1} & I\end{bmatrix}$$

where subscripts *c* and *n* stand for core and noncore animals, respectively; $\:\mathbf{M}=\:\mathrm{d}\mathrm{i}\mathrm{a}\mathrm{g}({\mathrm{g}}_{\mathrm{j}\mathrm{j}}-{\mathrm{g}}_{\mathrm{j},\mathrm{c}}{\mathbf{G}}_{\mathrm{c}\mathrm{c}}^{-1}{\mathrm{g}}_{\mathrm{c},\mathrm{j}})$, for the j^th^ noncore animal. In this population, 20,600 genotyped animals were randomly selected as core based on the number of largest eigenvalues explaining 98% of the variance in the spectrum of the genomic relationship matrix [21].

To assess potential reranking across ages, Spearman’s correlations between GEBV at different ages were computed, considering sires with at least one daughter with a record. Additionally, the percentage of sires selected in common across ages within the top 1% percentile was also calculated.

## Results and discussion

### Model comparison

Random regression models are currently regarded as the optimal method for the genetic evaluation of cow’s longevity [3, 22]. The predictive performance of RRM is largely determined by how well the model fits the data, which is influenced by the type and order of polynomials used [23]. In this study, contemporary group, additive genetic, and permanent environmental effects for functional longevity in an Angus population were modeled using orthogonal Legendre polynomials (LP), considering both homogeneous and heterogeneous residual variances.

The models were compared using the Deviance Information Criterion. The DIC balances two competing aspects: goodness-of-fit, which assesses how well the model explains the data, and model complexity, which penalizes models with excessive parameterization to avoid overfitting. A lower DIC value indicates a better balance between these two aspects, reflecting a more parsimonious and effective model. Table 2 presents the DIC values for the different models. The DIC decreased as the order of LP increased, indicating that higher-order LP models provided a better predictability. Models with heterogeneous residual variance, however, exhibited higher DIC values.

**Table 2:** Deviance information criterion (DIC) for different models with legendre polynomials

Residual Variance Classes	Leg1	Leg2	Leg3
1	1,436,846.51	498,209.35	50,500.39
9	2,438,376.35	4,194,257.63	6,953,927.00

LEGx, Legendre polynomial, in which x = 1, 2 and 3 is the order for the contemporary group, additive genetic and permanent environmental regressions

The best fit was observed with an LP of order 3 and homogeneous residual variance, yielding a DIC value of 50,500. This suggests that this model was identified as the most suitable for genetic evaluation, with no significant changes in residual variance over the cow’s lifespan. Due to the accumulation of annual performances in the phenotype, an increase in residual variance with age could theoretically be expected. However, because the residual variance was of small magnitude compared with the genetic variance and, especially, the permanent environmental variance, which increased considerably with age, accounting for heterogeneous residual variances did not improve model fit. The use of Legendre polynomials, compared to other types of polynomials [24, 25], aligns with most studies on beef cattle that employ RRM to evaluate cow fertility [3, 9, 10]. However, the optimal polynomial order and the number of residual variance classes are specific to the population and trait. Notably, this study introduces a novel functional longevity definition that considers the total number of calves per age, whereas most previous studies used binary records indicating whether the cow calved annually [9, 10]. This study corroborates Jamrozik et al. [9] who demonstrated that a model using Legendre polynomials of order 3 provided a better fit for longitudinal stayability data in Canadian Simmentals. In contrast, Silva et al. [10] found that models using Legendre polynomials of order 5 were superior for genomic prediction of stayability in the Nellore and Tabapua breeds, while an order 6 model was recommended for the Guzerat breed. Oliveira et al. [3] further indicated that an RRM based on Legendre of order 4 with heterogeneous residual variance was most suitable for assessing longitudinal binary functional longevity data in the Angus breed. This contrasts with the findings of Oliveira et al. [3] may be attributed to the trait definition. Unlike binary longevity traits, which can exhibit abrupt transitions and inflated residuals due to dichotomous outcomes, our cumulative definition yields a continuous and monotonically increasing trajectory across ages. This structure smooths inter-age variability and stabilizes residual variance, allowing the covariance structure to be effectively captured using lower-order polynomials. Consequently, a more parsimonious model (LP3 with homogeneous residuals) was sufficient to achieve optimal fit and predictive performance.

In addition to model comparison based on the DIC, we conducted a validation analysis using the LR method proposed by Legarra and Reverter [15] to assess prediction accuracy and bias across models. The validation was performed using GEBVs at age 6, which, as further discussed, proved to be the optimal reference point for selection. The results, presented in Table 3, reinforce the choice of the third-order Legendre polynomial model with homogeneous residual variance. This model yielded the highest prediction accuracy (0.42) and minimal bias (0.01), indicating a high predictive performance. While the bias estimates for the second- and third-order models were similar, the second-order model showed lower accuracy (0.25). In contrast, the first-order model exhibited both lower prediction accuracy (0.11) and higher bias (0.15), suggesting limited suitability for genomic evaluations. Taken together, the validation results support the Legendre 3 model as the most robust and predictive option for estimating functional longevity in Angus cattle.

**Table 3:** prediction accuracy ($\:\widehat{\boldsymbol{a}\boldsymbol{c}\boldsymbol{c}})$and bias ($\:{\boldsymbol{\mu\:}}_{\boldsymbol{w}\boldsymbol{p}}$) for different models with Legendre polynomials

Residual Variance Classes	Leg1		Leg2		Leg3	
	$\:\widehat{acc}$	$\:{\mu\:}_{wp}$	$\:\widehat{acc}$	$\:{\mu\:}_{wp}$	$\:\widehat{acc}$	$\:{\mu\:}_{wp}$
1	0.11	0.15	0.25	0.01	0.42	0.01
9	0.10	0.16	0.22	0.01	0.39	0.01

LEGx, Legendre polynomial, in which x = 1, 2 and 3 is the order for the contemporary group, additive genetic and permanent environmental regressions

Although the optimal model parameters may vary based on population and trait, the findings of this study contribute to expanding the knowledge on the application of RRM, further advancing our understanding of cow longevity and fertility.

### Heritabilities

**Fig. 2:**
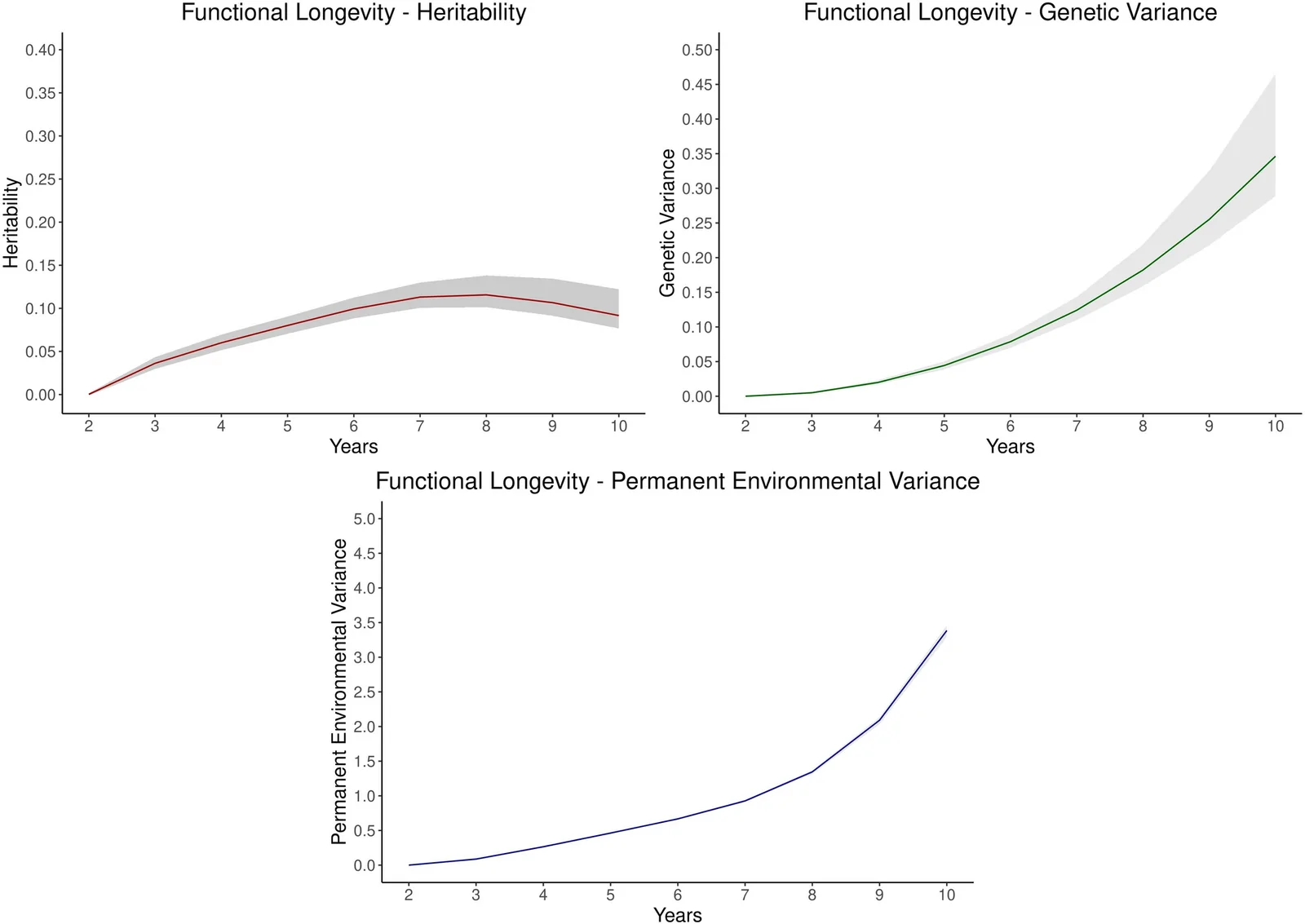
Posterior Mean Curves of Functional Longevity Genetic Parameters. Legend. Posterior means and 95% highest probability density intervals (HPD95) curves for heritability, genetic variance and permanent environmental variance over cow’s ages for functional longevity in Angus cattle

The posterior means of the genetic parameters and their corresponding curves for FL from ages 2 to 10 in the cows’ lifespans are presented in Table 4; Fig. 2, respectively.

**Table 4:** Posterior means and 95% highest posterior density intervals for genetic parameters of functional longevity across cow ages (2–10 years)

Ages	$\:{h}_{a}^{2}$	$\:{{\upsigma\:}}_{a}^{2}$	$\:{{\upsigma\:}}_{pe}^{2}$	$\:{{\upsigma\:}}_{e}^{2}$
2	0.000 (0.000–0.000)	0.000 (0.000–0.000)	0.000 (0.000-0.001)	0.046
3	0.036 (0.03–0.043)	0.005 (0.004–0.006)	0.088 (0.087–0.089)	0.046
4	0.060 (0.052–0.07)	0.020 (0.017–0.023)	0.265 (0.262–0.268)	0.046
5	0.080 (0.071–0.091)	0.044 (0.039–0.05)	0.463 (0.457–0.468)	0.046
6	0.099 (0.088–0.112)	0.079 (0.070–0.090)	0.668 (0.658–0.677)	0.046
7	0.113 (0.101–0.13)	0.124 (0.110–0.143)	0.927 (0.910–0.942)	0.046
8	0.116 (0.101–0.138)	0.182 (0.159–0.219)	1.346 (1.315–1.372)	0.046
9	0.107 (0.092–0.135)	0.255 (0.218–0.325)	2.091 (2.035–2.135)	0.046
10	0.092 (0.077–0.122)	0.346 (0.289–0.466)	3.385 (3.29–3.459)	0.046

h², heritability;$\:{{\upsigma\:}}_{a}^{2}$, additive genetic variance;$\:{{\upsigma\:}}_{\mathrm{p}\mathrm{e}}^{2}$, permanent environmental variance;$\:{{\upsigma\:}}_{e}^{2}$, residual variance

The FL genetic parameters changed over the cow’s age (Fig. 2), with generally low heritability estimates, increasing up to age 7, followed by a slight decrease thereafter. Despite the low heritability, direct selection for FL is feasible, but the annual genetic gain may be more limited than in traits with higher heritability. Given the economic importance of functional longevity to the beef cattle industry [1, 26], it is recommended to incorporate this trait into selection indices aimed at long-term selection.

Lower heritability values were observed between 2 and 5 years of age. The heritability of 0 at age 2 is likely due to the lack of variance in records, as all cows exhibit the same phenotype of 1 calving at this age. The lower heritability at age 3 may be attributed to the increased influence of non-genetic effect on female re-breeding after first calving. First-parity cows’ re-breeding is a major challenge in beef production due to nutritional limitations [27] and physiological demand simultaneously imposed by growth and lactation processes [28]. Under conditions of greater environmental instability, environmental effects are expected to dominate phenotypic variation, which helps explain the lower heritability estimates observed at early reproductive stages. This pattern is inherent to the dataset and trait definition, and reduced early-age heritability has been consistently reported in the literature [3, 4]. Modeling functional longevity (FL) as a cumulative trait enables the estimation of age-specific variances without inflating residual variance, which is a known concern in binary approaches [29, 30]. Additionally, the cumulative trait allows the model to more accurately capture the dynamic interplay between genetic and environmental factors over time, providing a more comprehensive representation of overall reproductive performance.

The highest heritability estimates were observed at older ages, with a peak at age 8 (0.12). However, based on the HPD95 interval, no significant differences in heritability estimates were detected between ages 6, 7, 8, 9, and 10. Therefore, selecting for FL at any of these ages will yield similar genetic gains. The FL heritability estimates of the present study (0.04 to 0.12) are, on average, lower than those reported by Oliveira et al. [3], who found heritability for binary functional longevity data ranging from 0.09 to 0.28. The observed differences may arise from variations in trait definition, modeling strategy, and population composition, all of which can directly influence estimates of genetic parameters. Nonetheless, as previously discussed, the cumulative approach enables a more detailed assessment of cow reproduction and provides a robust framework for characterizing the relationship between genetic and environmental variance.

The genetic and permanent environmental variances increased 69.2-fold and 38.4-fold, respectively, between ages 3 and 10. At age 10, the ratios of the genetic and permanent environmental variances to the residual variance, which was assumed to be homogeneous across ages, were 7.5 and 73.6, respectively. This pattern is a consequence of modeling FL as a cumulative phenotype. Because each yearly record represents the accumulation of previous reproductive outcomes, increases in these variance components across ages are expected.

### Genetic correlations

The (co)variance components across ages were derived from regression coefficients, and genetic correlations between different ages were estimated (Table 5). Since there is no variance at age 2, the following results exclude this age, as the prediction error variance can increase when contemporary groups exhibit no variance. This affects both the genetic parameter estimates and the prediction accuracy of expected progeny differences [31]. The correlations were positive and high, ranging from 0.86 to 1.00, with only minor fluctuations in magnitude throughout the cows’ lifetime. The strongest correlations were observed between adjacent years and were slightly weaker as the interval between years increased. This pattern suggests that selection for FL based on any given age would influence FL across a cow’s life, up to 10 years of age.

**Table 5:** Genetic correlations between cow’s ages for functional longevity in Angus cattle

Ages/Ages	3	4	5	6	7	8	9	10
3	1.00	1.00	0.99	0.97	0.95	0.93	0.90	0.86
4		1.00	1.00	0.99	0.97	0.95	0.93	0.90
5			1.00	1.00	0.99	0.97	0.96	0.93
6				1.00	1.00	0.99	0.98	0.96
7					1.00	1.00	0.99	0.98
8		Symmetric				1.00	1.00	0.99
9							1.00	1.00
10								1.00

The lowest genetic correlation was observed between ages 3 and 10 (0.86), indicating a gradual decline as the temporal gap between ages increases. This pattern suggests that distinct sets of genes may influence reproductive performance at different stages of a cow’s life. Such variation is likely driven by changing physiological demands throughout the lifespan, including shifts in energy allocation, maintenance requirements, and lactation intensity [27]. Nonetheless, the results suggest that, in general, the fertility of older cows is closely related to the reproductive efficiency of younger cows. This result corroborates the findings of Silva et al. [10] and Ramos et al. [4], who reported high correlations between stayability at the second calving and older ages in zebu populations. The same conclusion was drawn by Jamrozik et al. [9], who reported genetic correlations ranging from 0.74 to 0.99 between ages 3 and 8 years for stayability in the Simental breed, suggesting that selection could be performed at the second calving to reduce the generation interval and increase annual genetic gain.

Despite the high genetic correlation between early and later years, the present study shows that the early years are not ideal as indirect selection criteria under our proposed functional longevity definition. This is due to the lower heritability estimates observed at ages 2, 3, 4, and 5, which may result in slower genetic progress compared to later years. In contrast, ages 6 to 10 exhibited higher heritability estimates, with no significant differences, along with strong genetic correlations. This suggests that direct selection based on any of these years would result in similar genetic gains, positively influencing functional longevity at earlier ages.

### GEBV ranking of animals

The present study integrates RRM with single nucleotide polymorphism (SNP) data through the ssGBLUP method. Combining genomic information with RRM is particularly effective for enhancing prediction accuracy in young animals. The RRM framework accounts for individuals with limited longitudinal data by modeling the covariance structure across ages, enabling the early inclusion of young females in genetic evaluations. Incorporating genomic data further improves accuracy by capturing realized genetic relationships via the genomic relationship matrix, rather than relying solely on expected pedigree relationships. This integration is especially advantageous for traits such as longevity, which manifest later in life, exhibit low heritability, and are recorded only in females [32].

The GEBV ranking of sires with at least one daughter with records was assessed across ages using the Spearman correlation (Table 6) and the percentage of commonly selected individuals in the top 1% (Table 7). Overall, Spearman correlations were high, ranging from 0.84 to 1.00, indicating strong consistency in sire rankings across different ages.

**Table 6:** Spearman correlations between Functional Longevity GEBV of each evaluated age, considering sires with at least one daughter with records

Ages/Ages	3	4	5	6	7	8	9	10
3	1.00	0.99	0.98	0.96	0.94	0.91	0.88	0.84
4		1.00	1.00	0.99	0.97	0.95	0.92	0.89
5			1.00	1.00	0.99	0.97	0.95	0.92
6				1.00	1.00	0.99	0.97	0.95
7					1.00	1.00	0.99	0.97
8		Symmetric				1.00	1.00	0.99
9							1.00	1.00
10								1.00

**Table 7:** Percentage of commonly selected individuals for functional longevity based on different ages, considering the top 1% sires with at least one daughter with records

Ages/Ages	3	4	5	6	7	8	9	10
3	100%	87%	80%	74%	68%	61%	57%	53%
4		100%	92%	86%	79%	72%	67%	62%
5			100%	93%	86%	80%	74%	69%
6				100%	93%	86%	80%	75%
7					100%	93%	87%	82%
8		Symmetric				100%	94%	89%
9							100%	95%
10								100%

The percentage of commonly selected sires among the top 1% ranged from 53% to 95%. High correlations (above 0.98) and common selection percentages (above 87%) were observed between adjacent age groups, suggesting that selecting the top 1% of sires based on adjacent years of age would lead to the selection of a similar group of individuals.

The magnitude of the Spearman correlation slightly declined with increasing age gaps. The lowest correlation (0.84) was observed between the extreme ages of 3 and 10, indicating the highest variation in sire rankings. The percentage of selected individuals exhibited a pronounced decline with greater age differences, presenting a value of 53% observed between ages 3 and 10. This finding suggests that selecting the top 1% sires based on age 10 would result in a different group of animals when compared to age 3. The lower Spearman correlation and percentage of common individuals can be attributed to the reduced genetic correlation (0.86) between these two ages, as previously discussed.

With the model proposed for FL in this study, GEBV and EPD can be calculated at all time points, however, to ease the interpretation of results and facilitate the selection decision by Angus breeders, predicting EPD at an age point is a better approach. Given the heritability estimates, genetic correlation across years, and the average age of the cows in the data available (6 years old, on average), age 6 is the optimal reference point for publishing the genetic evaluation and for selection. Regardless of which time point the EPD are predicted, incorporating records from older ages can further improve prediction accuracy, as random regression models (RRM) account for the covariance structure between ages [7]. This aligns with the findings of Sánchez-Castro et al. [22], who showed that including older age records (e.g., 7 and 12 years) enhances the accuracy of EBVs for stayability at the traditional 6-year reference point in Angus cows (calculated according to the Beef Improvement Federation Guidelines [33]).

### Using cumulative versus binary phenotypes

Traditionally, cow longevity evaluations rely on binary phenotypes, where reproductive performance is assessed using categorical records (e.g., presence or absence of calving at a given age) [9, 10]. In contrast, the proposed approach defines FL as the total number of calves produced across ages, providing a more biologically meaningful representation of reproductive performance over time. By capturing the total reproductive output up to each specific age, this approach offers a more nuanced assessment of fertility, penalizing irregular calving intervals while more accurately reflecting a cow’s overall reproductive performance.

A key limitation of binary phenotypes in fertility evaluations is their constrained variation, which arises from the dichotomous nature of the trait definition. The high frequency of extreme values (zeros and ones) may inflate the residuals and increase the prediction error variances [29, 30]. Additionally, binary traits may rely on threshold models, which assume an underlying normal variable (liability) that maps onto the observed categories of the trait through fixed thresholds. Therefore, the liability is assumed to have a joint distribution with the observed phenotypes [8]. However, the nonlinear equations and normal probability functions inherent in threshold models introduce greater computational complexity and demand more computing resources compared to standard mixed models [29]. These computational challenges pose practical constraints for large-scale breeding programs that require efficient and timely genetic evaluations [8].

Additionally, in longitudinal genetic evaluations, RRMs assume residuals are uncorrelated across ages, a simplification that may overlook persistent environmental effects. This assumption is particularly problematic for repeated binary traits, where successive observations are often correlated [34]. In contrast, the cumulative definition represents a count trait with genuine and greater variation across ages, which helps reduce residual dependency and improves the robustness of the model.

The cumulative phenotype approach represents a significant methodological advancement by simplifying and potentially enhancing the genetic evaluation process. It directly incorporates the total number of calves produced, reflecting the cow’s overall reproductive efficiency at each specific age. In contrast, the binary approach usually evaluates each age separately, estimating the probability of a cow calving at each evaluated age. However, this probability may not fully capture cows with consistent parturition across multiple years, requiring a post-processing step to calculate the FL EPD, which reflects the reproductive performance over time.

Additionally, while the binary approach allows for the estimation of the probability of consecutive calvings by categorizing reproductive success or failure based on a cow’s ability to produce consecutive calves up to each evaluated age, it has a significant limitation: it inherently discards information from cows that experience reproductive interruptions. If a cow fails to calve in a given year, subsequent records are often treated as missing or failures, implying that she has failed to produce consecutive calvings and disregarding any later successful calvings. In such cases, the binary classification oversimplifies reproductive trajectories and may result in the misclassification of cows with non-consecutive calvings as functionally identical. In contrast, the cumulative approach preserves data from all recorded calvings, providing a more comprehensive dataset for genetic evaluations.

Another key advantage of the cumulative approach is its direct interpretability. Unlike threshold models, which require transforming EPDs from the liability to the probability scale, to provide a biological interpretation for selection, the cumulative measure directly reflects lifetime reproductive output. It enhances the clarity and practical utility of genetic evaluations, enabling breeders to make more informed selection decisions.

In summary, the adoption of the linear cumulative phenotypes definition for FL represents a significant advancement in genetic evaluation methodologies. By offering a more precise, interpretable, and computationally efficient approach, it overcomes the key limitations of binary phenotypes, providing a robust alternative for improving fertility selection in beef cattle breeding programs.

## Conclusions

The proposed definition of functional longevity, based on the total number of calves a cow has produced by each evaluated age under a RRM, provides a feasible and effective approach for a FL genomic evaluation in Angus cattle. A random regression model with homogeneous residual variance and third-order Legendre orthogonal polynomials is recommended for this trait and population. Genetic parameter estimates for functional longevity showed slight fluctuations throughout the cows’ lifespan up to 10 years of age. The heritability estimates indicate sufficient additive genetic variance to allow a moderate response to selection. Furthermore, based on genetic correlations and ranking comparisons, age 6 is identified as the optimal reference point to maximize selection response for improved longevity in North American Angus cattle.

## Data Availability

The data cannot be shared since it belongs to the American Angus Association (Saint Joseph, MO).
